# Use of Integrase-Minus Lentiviral Vector for Transient Expression

**Published:** 2012-08-31

**Authors:** Touraj Farazmandfar, Hossein Khanahmad Shahreza, Mohammad Reza Haghshenas, Ghasem Janbabai, Hossein Azadeh, Nader Mansour Samaei

**Affiliations:** 1. Faculty of Advanced Medical Science Technology, Golestan University of Medical Sciences, Gorgan, Iran; 2. Molecular and Cell Biology Research Center, Mazandaran University of Medical Sciences, Sari, Iran; 3. Cancer Research Center, Mazandaran University of Medical Sciences, Sari, Iran; 4. Pasteur Research and Production Complex, Karaj, Iran

**Keywords:** Lentiviral Vector, Integrase-Minus, Transient Expression

## Abstract

**Objective:**

Lentivirus-derived vectors are among the most promising viral vectors for gene therapy which is currently available, but their use in clinical practice is limited due to associated risk of insertional mutagenesis. Gene targeting is an ideal method for gene therapy, but it has low efficiency in comparison to viral vector methods. In this study, we are going to design and construct an integrase-minus lentiviral vector. This vector is suitable for transient expression of gene and gene targeting with viral vector.

**Materials and Methods:**

In this experimental study, three missense mutations were induced in the catalytic domain of Integrase gene in the *pLP1* plasmid and resulted *D64V*, *D116A* and *E152G* changes in the amino acid sequence through site directed mutagenesis. The *pLenti6.2-GW/EmGFP* transfer vector, associated with native and mutated packaging mix, was transfected into *293T* cell line. In order to titer the lentivirus stock, the viruses were harvested. Finally, the viruses transduced into *COS-7* cell line to assess green fluorescent protein (GFP) gene expression by a fluorescence microscopy.

**Results:**

Recombinant and wild lentiviruses titer was about 5~8×10^6^ transducing units/ml in *COS-7* cell line. The number of GFP-positive cells transduced with native viruses was decreased slightly during two weeks after viral transduction. In contrast, in the case of integrase-minus viruses, a dramatic decrease in the number of GFP positive cells was observed.

**Conclusion:**

This study was conducted to overcome the integration of lentiviral genome into a host genome. Nonintegrating lentiviral vectors can be used for transient gene expression and gene targeting if a Target gene cassette is placed in the lentivirus gene structure. This combination method decreases disadvantages of both processes, such as random integration of lentiviruses and low efficiency of gene targeting.

## Introduction

Vectors based on retroviruses and lentiviruses have been used to introduce genes efficiently and stably into cells for long-term expression. Their ability to stable transduces in cells is due to encode integrase protein in their genomes. This protein identifies specific sequences called long terminal repeats (*LTR*) in the virus genome and led to the insertion of these sequences in the host chromosomes (1, 2). Lentiviral vectors can deliver up to 8 kb of DNA of interest. They can be pseudotyped, so they display the chosen tropism. In addition, the lentiviral vectors can transduce dividing cells and non-dividing cells (3, 4), a great advantage for genetically modifying tissues, such as brain, muscle, liver, lungs, and hematopoietic system. They are easily produced without need of helper particles, and they are only weakly immunogenic (5, 6). Lentiviral vectors, like all integrative viral vectors, represent a risk of insertion mutations, which limits their use in clinical applications.

The third-generation Lentiviral Expression System is based on vectors developed by Dull et al. (7). This system has a significant number of safety features, including:

1. Development of a deletion in the 3′ *LTR* (ΔU3) that does not affect the generation of the viral genome in the producer cell line, but results in self-inactivation (SIN) of the lentivirus after transduction of the target cell (8).
2. Reduction of genes to three (*i.e. gag*, *pol*, and *rev*).
3. Usage of the *VSV-G* gene in place of the human immunodeficiency virus (HIV) envelope for production of a high titer lentiviral vector with a significantly broadened host cell range (9).
4. Insertion of genes encoding the structural and packaging components required for making virus into four separate plasmids (7).

In this generation of vectors, none of the HIV structural genes are actually present in the packaged viral genome, thus they are never expressed in the transduced target cell. So, no new replication-competent virus can be produced.

Although the integrated form of lentivirus DNA is classically considered to be responsible for viral gene expression, several studies have suggested that nonintegrated viral DNA can support transcription (10, 11). Themis et al. (12) have reported the oncogenic potential of lentiviral vectors after the transduction of fetal and neonatal tissues. These observations have led to the development of several integrase-defective [Int(-)] lentiviral vectors (13, 14). These vectors were made of specific mutations designed to defuse vector integration without affecting vector entry into cells, providing short-term gene expression. Lentivirus integrase is a 32-kDa protein with a core domain that contains a triad of amino acids essential for its catalytic activity, specifically aspartic acid 64, aspartic acid 116, and glutamic acid 152 (15-17).

The aim of this study was to design nonintegrating lentiviral vector by mutating aspartic acid 64 to valine (*D64V*), aspartic acid 116 to alanine (*D116A*) and glutamic acid 152 to alanine (*E152A*). We followed the levels of expression of the green fluorescent protein (GFP) reporter gene from native and nonintegrating vectors. These vectors can be used for transient gene delivery and gene targeting with this feature.

## Materials and Methods

### Construction of lentiviral vector

In this expereimental assay, lentiviral particles was produced using* pLenti6.2-GW/EmGFP* Transfer vector (Invitrogen Corporation, Grand Island, NY, USA) contained the Emerald GFP reporter gene and the human *CMV* promoter with the third-generation ViraPower included three helper plasmids, as: *pLP1*, *pLP2*, and *pLP/VSVG* (Invitrogen Corporation, Grand Island, NY, USA). These helper plasmids supply, structural, and replication proteins required to produce functional virus. At first, the Int (-) packaging plasmid *pLP1* was constructed using three missense mutations in active sites of the integrase catalytic domain. The *D64V*, *D116A* and *E152G* changes were made in the amino acid sequence with the site directed mutagenesis method by primers: *D64V* Forward 5'-AGCTAGTATGtaCACATTTAGAAGG-3', *D64V* Reverse 5'-TTCTAAATGTGTACAtaCTAGCTGC-3', *D116A* Forward 5'-AGTACATACAGcaAATGGCAG-3' , *D116A* Reverse 5'-TGCCATTtgCTGTATGTACTG-3', *E152G* Forward 5'-GTCAAGGAGTAATAGctTCTATG-3', *E152G* Reverse 5'-CTTTATTCATAGAagCTATTACTC-3'.

### Producing lentivirus in 293T cells

The new generated Int(-) *pLP1* and three other plasmids, including *pLenti6.2-GW/EmGFP, pLP2* and *pLP/VSV-G* were amplified, then their concentrations was adjusted to 1 µg/µl. 293T cells (ATCC No. CRL-11268) reachedare grown to 90% confluency in two 100 mm dishes. Dishes were transfected with the followings: 6 µg *pLP1* plasmid [Int (-) *pLP1* was used in order to prepareto make the Integrase-Minus virus and native *pLP1* to make integrating virus], 14 µg *pLenti6.2-GW/EmGFP*, 2.4 µg *pLP2* and 3.4 µg *pLP/VSV-G* with 50 µl Lipofectamine 2000 (Invitrogen Corporation, Grand Island, NY, USA), and 2.5 ml serum-free Dulbecco’s modifi ed Eagle’s medium (DMEM) (GIBCO, Frankfurt Germany). The cells were then incubated 4-6 hours at 37℃ in a CO_2_ incubator. It was followed by adding 3 ml DMEM supplemented with 10% fetal bovine serum (FBS) (GIBCO, Frankfurt Germany), 100 IU/ml penicillin, 2 mmol/L L-Glutamine and 100 µg/ml Streptomycin (Invitrogen Corporation, Grand Island, NY, USA)., Then, the mixture was left overnight at 37℃ in a CO_2_ incubator. The supernatants were changed and harvested every day for the next 3-4 days. The supernatants were centrifuged at 3000 rpm for 15 minutes at +4℃ to obtain pellet debris. The viral supernatants were transferred through pipet into cryovials in 1 ml aliquots and stored them at -80℃.

### Titering lentiviral stock

Viral vector titers were assayed using Invitrogen’s ViraPower protocol by infection of *293T* cells at different dilutions (10^2^-10^6^) in a 6-well plate (one mock well plus five dilutions) for every lentiviral stock (mutant and native). The titers were 5~8×10^6^ transducing units (TU)/ml and calculated by counting GFP-positive cells in two different dilutions with countable number of cells using a fluorescence microscopy (NIKON, Japan).

### Transduction of COS7 cells

The African green monkey cell line, COS-7 (ATCC No. CRL-1651), was thawed and expanded in DMEM (10% FBS, 100 u/ml penicillin, 100 Ag/ml streptomycin, and 2 mmol/liter L-glutamine) to 1×10^6^ cells/ml in culture flasks. Cells were then treated with vector at an multiplicity of infection (MOI) of 2 in 500 µl for 2 hours in the presence of 80 mg/ml polybrene and expanded to total volume of 2 ml. Cells were analyzed for GFP expression by a fluorescence microscopy.

The Mazandaran University of Medical Sciences Research Ethics Committee approves this study from an ethical point of view.

## Results

All integrase-minus vectors gave titers within 48 hours and they were comparable to those of the native vector. It indicated that the mutations made to create integrase-minus vectors did not affect their ability to produce functional virus particles.

**Fig 1:**
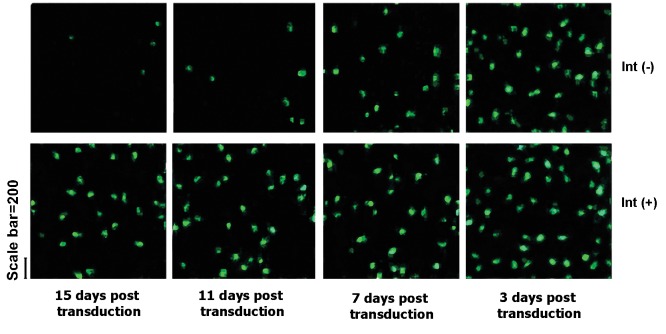
*GFP* expression in dividing cells. The *COS7* cells were transduced with equivalent *TU* amounts of the Int(-) or the native vectors. Cells were cultured and analyzed by a fluorescence microscopy at different time interval after transduction to determine the percentage of *GFP*-positive cells.

**Fig 2:**
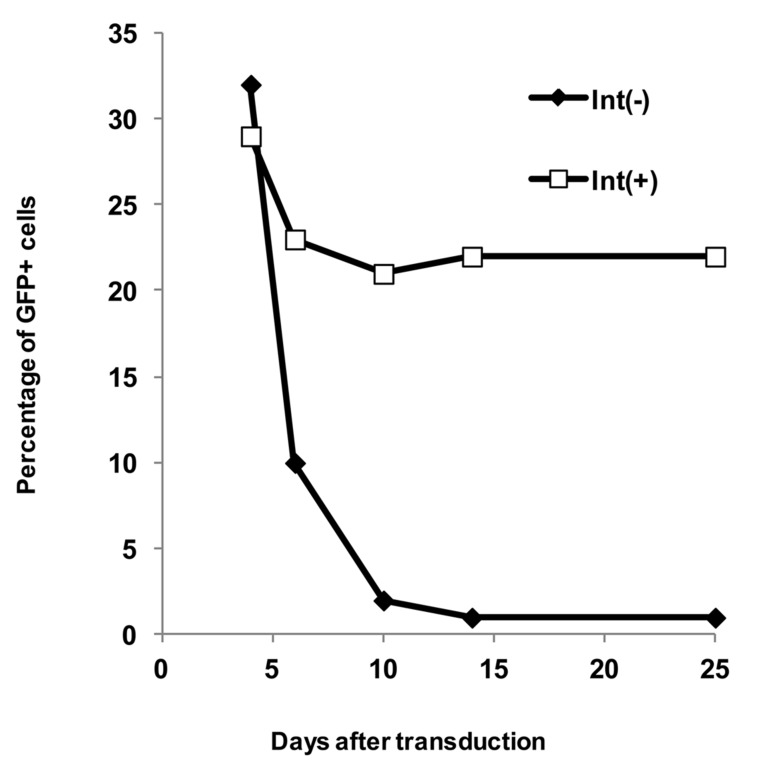
*GFP* expression in *COS7* cells *in vitro*. *GFP* expression in *COS7* cells was evaluated after transduction with equal TU amounts of Int(-) or native vectors. The percentage of *GFP*-expressing cells was evaluated up to 20 days.

We confirmed that GFP expression from integrase-minus vectors did not result from integrated provirus because GFP fluorescence levels decreased during the consecutive passages. Cells were initially transduced with equal transduction unit (TU) amounts of vector (mutant and native), then and cultured and passaged for up to 20 days. The cells were consistently analyzed by a fluorescence microscopy to assure of stability assessment of GFP expression through passages (Fig 1) . The percentage of GFP-positive cells was stable after transduction with the integrative vectors. Also, its growth rates were 28.42 ± 0.22% and 22.64 ± 1.25% after 3 and 20 days, respectively. In contrast, the percentage of GFP fluorescence cells were rapidly decreased in cells transduced with the mutant integrase-minus vectors, and its growth rates were 31.81 ± 0.36% and 0.7 ± 0.09% after 3 and 20 days, respectively (Fig 2). Progressive loss of transgene expression in dividing cells observed with the recombinant vector was consistent with a nonintegrating phenotype.

## Discussion

In this study, we constructed nonintegrating lentiviral vectors for high efficiency gene transfer to primary cells for transient gene expression. We produced integrase-defective vectors from HIV-1-based lentiviral vectors by introducing mutations to inactivate the integrase catalytic function in the viral genome. Applying a packaging plasmid with missense mutations in the integrase gene was highly effective to limit constant expression. In a standard packaging plasmid, the integrase gene open-reading frame needs to maintain in order to allow sufficient DNA synthesis, so only few missense mutations, none of nonsense mutation or large deletion, can be used (18). The rare integrations created by nonintegrating vectors may also occur with other transient gene delivery methods, such as adenoviral vectors and nonviral plasmid-mediated gene delivery. The transduction efficiency of the *D64V* vector reported by Yanez-Munoz et al. (19) was equal to the integrative vector; whereas, the mutant vector described in this study showed lower transduction efficiency. Although further comparison studies are needed, this difference may be explained by catalyzing integration reactions. Integrase enzyme is involved in various steps of the virus life cycle. Thus, the integrase gene mutations used in this study may impair transduction efficiency. We developed an integrase-minus lentiviral vector that was nonintegrating and allowed the formation of circular episomal genomes in the nucleus of transduced cells. We showed that this nonintegrated form was efficiently transcribed by the cellular machinery. However, transgene expression with the recombinant vector did not appear to be as efficient as its native counterpart. These newly developed Int(-) vectors illustrated a step forward in the clinical application of lentiviral vectors for gene delivery. Since these vectors retain very weak and insignificant in integration activity, the risk of insertional mutagenesis is totally canceled. Integrase-minus lentiviral vectors can be used for gene targeting if a Target gene cassette is placed in the lentivirus gene structure. This method decreases disadvantage of low efficiency of gene targeting.

## Conclusion

These vectors could be used in gene therapy methods inquiring transient expression of transgene, in dividing cells, and long-term gene expression in non-dividing cells.
